# Radioguided surgery in urological malignancies

**DOI:** 10.4103/0970-1591.38595

**Published:** 2008

**Authors:** Dorothea Weckermann, Mark Thalgott, Gabriele Holl, Theodor Wagner, Rolf Harzmann

**Affiliations:** Department of Urology, Klinikum Augsburg, Germany; 1 Department of Nuclear Medicine, Klinikum Augsburg, Germany; 2 Department of Pathology, Klinikum Augsburg, Germany

**Keywords:** Bladder cancer, lymph node dissection, penile cancer, prostate cancer, radioguided surgery, renal cell cancer, sentinel lymph node, testicular cancer

## Abstract

The current literature was reviewed for articles focusing on radioguided surgery in urological malignancies.

In penile cancer sentinel lymph node dissection is part of international guidelines. By detailed histopathological analysis (serial sections, immunohistochemical staining) more micrometastases are detectable improving the histopathological staging.

In prostate cancer this technique also improves staging since a high percentage of patients have lymph node metastases located outside the region of standard lymphadenectomy. Compared to extended lymph node dissection radioguided surgery has a lower morbidity, especially a lower rate of lymphoceles.

In bladder cancer the sentinel lymph node (SLN) technique has some limitations. Combined with extended lymph node dissection more positive lymph nodes are removed which possibly improves survival.

In renal cell and testicular cancer there are only preliminary results. Further investigations will show whether this technique will play an important role in the diagnostics and therapy of these tumors.

In all urological malignancies the SLN concept is only a staging procedure. When the sentinel node(s) is (are) negative, the other lymph nodes are negative, too. Since there are no randomized prospective trials comparing the results of sentinel lymphadenectomy with other techniques of lymph node dissection, it is not clear whether sentinel lymph node dissection also has a prognostic impact.

Lymph node dissection in urological malignancies plays an important role. In contrast to preoperative imaging it renders an exact lymph node staging in order to calculate the risk of progression and to plan an appropriate adjuvant therapy. In addition to that the removal of lymph nodes which contain minimal metastatic disease could improve survival.

In 1960 Gould introduced the term sentinel lymph node and defined it in relation to a constant anatomical position in tumors of the parotid.[1] Sentinel lymph node detection was first achieved by performing lymphangiography but it was later done using different tracers, which changed the purely anatomical definition to a functionally identified lymph node with an inconsistent anatomical localization that is unique in each patient. Extended serial sectioning and immunohistochemical staining of SLN along with the increased detection of micrometastases (ultrastaging) has led to further development of this concept.[2]

## SLN DISSECTION IN PROSTATE CANCER

Homogeneous surgical standards of pelvic lymph node dissection in prostate cancer cannot be found in the current literature. Therefore, the percentage of pN+-stages distinctly varies depending on the treated patients and the extent and technique of lymphadenectomy. The minimal lymph node dissection only considers lymph nodes in the obturator fossa, whereas the standard lymphadenectomy also includes lymph nodes along the external iliac vessels. The extended lymph node dissection is a complete lymph node dissection along the external iliac vein, the internal iliac artery up to the common iliac vessels including presacral lymph nodes and lymph nodes in the obturator fossa. With the extended lymph node dissection more lymph nodes are removed and more lymph node metastases are detected. But, this technique requires much more time and has a higher complication rate than standard lymphadenectomy. Sentinel lymph node dissection could be a way out of this dilemma.

The Augsburg group first developed and described the SLN technique in prostate cancer.[3] Meanwhile, other groups from Austria,[4,5] France,[6,7] Italy,[8] Japan[9,10] and Brazil[11] confirmed the validity of this technique [Table 1]. In contrast to malignant melanoma and breast cancer the number of SLN is higher. This is predominantly caused by the fact that lymphatic drainage of the whole prostate and not of the tumor as a part of the organ is depicted. In prostate cancer lymphatic pathways are mostly absent, because of branching of pelvic lymphatic vessels and the low radioactivity which is arriving in the SLN. Therefore, it is impossible to demonstrate for every single radioactive lymph node, whether it is a primary draining lymph node or a subordinate.[12,13]

**Table 1:** The sentinel lymph node concept in prostate cancer (review of the literature)

Authors	patients (n)	patients with pN+ (%)	patients with pSLN+ (%)	localization of pSLN+
Bastide *et al.*,[6]	34	4/34 (11.8)	3/34 (8.8)	In two of four cases metastases were detected outside the region of standard lymph node dissection
Brenot-Rossi *et al.*,[7]	27	4/27 (14.8)	4/27 (14.8)	In two of four patients metastases were located in the region of the internal iliac artery
Corvin *et al.*,[17]	28	7/28 (25)	7/28 (25)	Three of 10 lymph node metastases were located outside the obturator fossa
Fukuda *et al.*,[10]	42	13/42 (31)	12/42 (28.6)	In seven patients metastases were located outside the region of standard lymphadenectomy
Jeschke *et al.*,[5]	140	19/140 (13.6)	19/140 (13.6)	71.4% of metastases were located outside the obturator fossa
Rudoni *et al.*,[8]	48	5/48 (10.4)	5/48 (10.4)	In two of five cases metastases were located outside the region of standard lymphadenectomy
Silva *et al.*,[11]	23	3/23 (13)	2/23 (8.7)	Two of three patients had metastases outside the obturator fossa
Takashima *et al.*,[9]	24	3/24 (12.5)	3/24 (12.5)	In all men metastases were located outside the region of standard lymph node dissection.
Weckermann *et al.*,[15]	1055	207/1055 (19.6)	205/1055 (19.4)	63.3% of men had metastases outside the region of standard lymphadenectomy

pN+ positive lymph nodes, pSLN+ positive sentinel lymph nodes

The technique of radioguided surgery in prostate cancer was first described by Wawroschek *et al.*[3] One day prior to lymphadenectomy technetium-99m nanocolloid is applicated transrectally into the prostate under ultrasound guidance. This tracer with a particle size of 100 nm or less is reliably transported from the interstitium to the initial lymph vessels and shows reproducible lymph node uptake.[14] Two hours after injection, scintigraphies in anteroposterior and dorsal projection are carried out. The lymphoscintigraphy shows the minimal number of radioactive lymph nodes which have to be detected intraoperatively. Furthermore, this technique allows one to locate obscured and surgically difficult accessible lymph nodes intraoperatively. During surgery radioactivity of lymph nodes is measured by gamma probes.

In 2007, the Augsburg group reported on SLN dissection in 1055 patients. Out of these 207 (19.6%) men had positive lymph nodes. In 205 men the SLN were positive. In 63.3% of men lymph node metastases were detected outside the region of standard lymphadenectomy. Eighty-two out of 207 men had only micrometastatic disease (ø 0.2 -2 mm). In 37 cases these micrometastases were only detected by immunohistochemical staining.[15] The results confirm that SLN dissection in prostate cancer has a high sensitivity in detecting positive nodes. When the SLN is/ are negative, the other pelvic lymph nodes are negative too in a high percentage of men (sensitivity 97.1%). Forty-two of 205 men with positive SLN (20.5%) had positive non-SLN, additionally. The higher the preoperative PSA, the pathological stage and Gleason score, the greater the percentage of men with positive SLN and positive non-SLN. This is the limitation of this technique.[16]

Meanwhile, the Augsburg group used the SLN technique in more than 1600 men with clinically organ-confined prostate cancer (unpublished data). No SLN were detectable intraoperatively in 38 out of 1698 men. Main reasons for this failure were neoadjuvant hormone therapy for more than six months and a preceding transurethral resection or adenomectomy of the prostate. Out of the remaining 1660 men 302 had positive lymph nodes (18.2%). In 297 men the SLN were positive. Only five out of 302 lymph node positive men had false negative results i.e. negative SLN and positive non-SLN (false negative rate 1.7%). Possible reasons for false negative results are macrometastases in SLN which destroy the normal architecture of the sentinel node. In these cases the radioactive tracer cannot be stored.

Corvin and Janetschek showed that SLN dissection is also possible laparoscopically.[4,17] Jeschke *et al.*, performed laparoscopic SLN dissection and radical prostatectomy in 140 patients with clinically localized prostate cancer. In eight men no SLN was detectable (5.7%). Out of the detected lymph node metastases 71.4% were located outside the obturator fossa.[5]

Bastide and Brenot-Rossi also studied the feasibility of SLN technique in prostate cancer. They detected SLN located along the hypogastric artery in a high proportion of men (77.8%).[6,7] Rudoni *et al.*, confirmed that SLN are often located at unusual sites compared to conventional lymph node dissection.[8] The Japanese group revealed that sensitivity and specifity of hot node prediction of lymph node metastases were 92.3 and 100%, respectively.[9,10] Silva *et al.*, from Brazil confirmed that SLN dissection adds important information to the staging of patients, not always attained through standard lymphadenectomy.[11]

Since the removal of sentinel lymph nodes has a low morbidity, especially a low rate of lymphoceles, this technique could be performed in all men with clinically organ-confined prostate cancer. In contrast to preoperative imaging and nomograms this technique renders an exact lymph node staging in every patient.

Häcker *et al.* investigated whether preoperative [^18^F] fluorocholine positron emission tomography-computerized tomography (PET-CT) and intraoperative laparoscopic radioisotope-guided sentinel lymph node dissection can detect pelvic lymph node metastases as reliably as extended pelvic lymph node dissection in men with clinically localized prostate cancer. In 10 out of 20 patients lymph node metastases were detected. [^18^F] fluorocholine PET-CT was true positive in one, false positive in two, false negative in nine and true negative in eight patients. The largest lymph node metastasis not seen with [^18^F] fluorocholine PET-CT was 8 mm.[18]

The current literature shows that SLN technique in prostate cancer is an excellent staging method. Since there are no randomized prospective studies comparing the results of no lymph node dissection with different techniques of lymphadenectomy, it is not clear whether the removal of SLN which contain minimal metastatic disease has an impact on survival.

## SENTINEL LYMPH NODE DISSECTION IN BLADDER CANCER

In bladder cancer the nodal status in addition to pathological stage represents the most important predictor of outcome of radical cystectomy in patients with muscle-invasive disease. But there is still considerable controversy regarding the appropriate extent of lymphadenectomy and the number of lymph nodes that should be dissected. Sherif *et al.*, performed the first study of SLN dissection in bladder cancer. In this pilot study a total of 13 patients who met the criteria qualifying them for radical cystectomy had intravesical injections of radioactive tracer and blue dye marker around the tumor followed by lymphoscintigraphy to visualize lymphatic drainage and detect sentinel nodes. Sentinel lymph nodes were identified in 85% (11/13) patients.[19]

Meanwhile, Liedberg and Månsson have greatest experience in SLN dissection in bladder cancer. In 2006, they reported 75 patients with invasive bladder cancer who underwent radical cystectomy with extended lymphadenectomy. Of 75 patients 32 (43%) were lymph node positive, of whom 13 (41%) had all lymph node metastases located only outside of the obturator spaces. An SLN was identified in 65 of 75 patients (87%). In seven patients the SLN was recognized when the nodal basins were assessed with the gamma probe after lymphadenectomy and cystectomy. Of the 32 lymph node positive cases 26 had positive (metastatic) SLN. Thus, the false negative rate was 19% (six of 32 cases). Five false negative cases had macrometastases and/or perivesical metastases. In nine patients the SLN contained micrometastases, in five of whom the micrometastases were the only metastatic deposit.[20]

In bladder cancer the SLN technique has some limitations. Patients with a large or multifocal tumor should be excluded because the radioactive tracer cannot be injected exactly around the tumor. Patients with macrometastasis in preoperative imaging should be also excluded, because in macrometastasis the tracer is not reliably stored (false negative SLN). If considering the limitations of this technique, SLN dissection is useful in bladder cancer, because it improves the detection rate of micrometastases. Because of the high false negative rate, it is not advisable to omit the extended lymphadenectomy in patients with muscle-invasive disease.

## SENTINEL LYMPH NODE DISSECTION IN RENAL CELL CANCER

In 1969, Robson claimed that extended lymph node dissection is necessary in the operative treatment of renal cell carcinoma (RCC).[21] Meanwhile, several investigations have shown that patients without suspicion of nodal metastases seem not to have any benefit regarding the overall outcome in comparison to sole radical nephrectomy (78% *vs.* 79%).[22] This is supported by the fact that only 3.3% of patients with unsuspicious preoperative staging show unexpected nodal metastasis during surgery.[23]

Recently, an improved survival has been demonstrated in patients with regional lymph node metastasis after lymph node dissection when nephrectomy is performed in combination with lymph node dissection due to a better postoperative response to immunotherapy.[24]

Bernie *et al.*,[25] tested the feasibility of SLN dissection in a porcine model injecting blue dye and 99m technetium nanocolloid into the kidney. Within 10 min the SLN harbored higher radioactive counts compared to controls and the radioisotope tracer did not enter the venous circulation. This study shows the feasibility of SLN dissection in an animal model, but it has still to be proven that this model of SLN dissection is also feasible in clinical trials and able to solve the controversies regarding the indication and extension of lymph node dissection in RCC.[26]

## SENTINEL LYMPH NODE DISSECTION IN PENILE CANCER

The squamous cell carcinoma of the penis metastasizes essentially via the subcutaneous lymphatic system by embolization from the superficial to the deep inguinal lymph nodes and lastly to the pelvic nodes without missing the previous compartment.[27,28]

About 58% of patients with penile cancer are diagnosed with palpable inguinal lymph nodes due to nodal metastases (17-45%) or inflammatory disease (55-83%).[29] But also patients without palpable lymph nodes reveal occult (micro)metastases in 16-73% depending on risk factors.[29]

It is generally accepted that the involvement of the lymphatic system is the most important prognostic factor for survival. Patients with negative lymph nodes have a five-year survival rate of 66%, as compared to 27% for patients with pN+ disease.[29] This fact is underlined by a five-year survival rate of nearly 100% in patients with negative lymph nodes who underwent a prophylactic lymph node dissection.[30]

Since groin dissection is associated with high morbidity and mortality rates (30-50% and 3%)[26] and about 82% of patients display negative nodes at prophylactic lymph node dissection and seem to be over-treated in this way,[31] there have been many controversies regarding the necessity and extension of inguinal lymph node dissection in clinically node-negative patients with penile cancer.

In 1977, Cabanas proposed the SLN biopsy based on the anatomical drainage of the penis by performing lymphangiography via the dorsal penile lymphatics. In all cases, the SLN was located within 1 cm of the superficial epigastric vein, showing that 12 of 15 patients with positive SLN had no further disease.[27] Since the anatomical approach does not take into account the individual drainage of the tumor, false negative results were subsequently reported in up to 25% (range 9-50%), concluding that the SLN biopsy as described by Cabanas was not further recommended.[32]

The technique of SLN injection does vary. Some authors inject patent blue dye 10-15 min before surgery intradermally around the tumor in addition to radiolabeled nanocolloids which are injected in the same way 4 h in advance.[26] The SLN detection happens by using a dual-head gamma camera as well as a hand-held gamma probe while the patent blue dye staining guides the dissection. Kroon and Horenblas reported 123 patients with ≥T2 penile cancer and non-palpable lymph nodes. In 23% (28/123 men) SLN harbored metastases, but the false negative rate was 18%. In the following time, this rate was reduced by routinely using preoperative ultrasound-guided fine needle aspiration cytology and performing inguinal lymph node dissection in case of positive lymph node biopsy or cytology.[33,34] All groins with no SLN visualized on lymphoscintigraphy were explored and wound palpation was now conducted intraoperatively. All SLN specimens were now subjected to serial sectioning and immunohistochemical staining. Subsequently, the false negative rate decreased.[26,33,34]

The technique of dynamic SLN biopsy in penile cancer is the only one that is recommended in international guidelines. It demonstrates a specificity of 100% and a sensitivity of 78-80%.[29] It is validated in many centers.[35] The technique itself is minimally invasive compared to prophylactic groin dissection, is not difficult to perform and it decreases the post interventional morbidity to 7%.[36] In SLN negative patients the five-year disease-specific survival rate was 96% in comparison to 66% in the SLN positive group.[37]

According to the European Association of Urology (EAU) guidelines on penile cancer the SLN technique is advisable for patients with low and intermediate disease defined as ≤T1 G2 with non-palpable lymph nodes, indicating modified or radical lymph node dissection if negative predictive factors like nodular growth, vascular invasion or positive dynamic sentinel node biopsy are present whereas cases with palpable lymph nodes should undergo radical lymph node dissection.[29] In case of macrometastatic involvement the lymphatic drainage may divert, marking another lymph node than the infiltrated SLN. This is the reason why this method is not recommended for patients with palpable lymph nodes.[26,33]

A recent series with 100 patients treated according to the EAU guidelines showed an overall survival rate of 92% demonstrating 18% lymph node involvement in non-palpable lymph node disease with a clear benefit for early lymph node dissection in men with positive nodal disease. On the other hand, being limited in predicting micrometastatic disease, 82% of patients underwent unnecessary prophylactic lymph node dissection with high morbidity.[31,38,39]

The Augsburg group evaluated the results of the first 11 patients with penile cancer who underwent SLN dissection. Tumor stages ranged from pT1G2 (n = 4) to pT3G2 (n = 7) displaying negative lymph nodes in the first group and six positive lymph nodes in the remaining seven patients of whom five men revealed only the SLN as affected. Up to now no recurrence has been observed during the follow-up.

## SENTINEL LYMPH NODE DISSECTION IN TESTICULAR CANCER

The greatest experience in SLN dissection in testicular cancer have Ohyama and Satoh from Japan,[40,41] who investigated 22 patients with Stage I disease. One day before surgery 99mTechnetium-labeled phytate was injected around the tumor. After radical orchiectomy gamma probe-guided laparoscopic retroperitoneal lymph node dissection was performed. The SLNs were detectable in 95% of patients. This concept was confirmed by Tanis *et al.*[42]

The data indicate the feasibility of SLN identification in Stage I testicular cancer. Further trials are still required to establish the SLN concept in this disease.
